# Y-DWMS: A Digital Watermark Management System Based on Smart Contracts

**DOI:** 10.3390/s19143091

**Published:** 2019-07-12

**Authors:** Bo Zhao, Liming Fang, Hanyi Zhang, Chunpeng Ge, Weizhi Meng, Liang Liu, Chunhua Su

**Affiliations:** 1 College of Computer Science and Technology, Nanjing University of Aeronautics and Astronautics, NO. 29 Yudao Street, Nanjing 210016, China; 2 Key Laboratory of Computer Network Technology of Jiangsu Province, Nanjing 210096, China; 3 Department of Applied Mathematics and Computer Science, Technical University of Denmark, 2800 Kongens Lyngby, Capital Region, Denmark; 4 Division of Computer Science, University of Aizu, Aizuwakamatsu 965-8580, Japan

**Keywords:** digital rights management, smart contract, blockchain, game theory

## Abstract

With the development of information technology, films, music, and other publications are inclined to be distributed in digitalized form. However, the low cost of data replication and dissemination leads to digital rights problems and brings huge economic losses. Up to now, existing digital rights management (DRM) schemes have been powerless to deter attempts of infringing digital rights and recover losses of copyright holders. This paper presents a YODA-based digital watermark management system (Y-DWMS), adopting non-repudiation of smart contract and blockchain, to implement a DRM mechanism to infinitely amplify the cost of infringement and recover losses copyright holders suffered once the infringement is reported. We adopt game analysis to prove that in Y-DWMS, the decision of non-infringement always dominates rational users, so as to fundamentally eradicate the infringement of digital rights, which current mainstream DRM schemes cannot reach.

## 1. Introduction

Digital publishing adopts a binary form to perform the storage and distribution of common publications (videos, music, images, etc.). Digital publications are valid without physical carriers of information, compared with common publications, which leads to a low cost of edition, replication, and distribution. However, this also invokes tough problems of digital rights management (DRM) [1]. For example, the film Wandering Earth released in China this year lost more than 140 million box office dollars during the first week of its release, due to unauthorized spreading of film copies.

DRM issues are diverse and complex, but we believe that the essence of digital copyright is value exchanging between copyright holders and users, while the essence of infringement is the imbalance between the profit of copyright holders and the value obtained by users. Then, infringers may perform infringement of copyright at any link in the flow of value. According to the common means infringers may take, the threat model in DRM could be defined as follows:

- An infringer obtains the copy of publication, refusing to pay copyright holders enough. Such scenarios include stealing a copy of publication, accessing it free via a third party, or purchasing it for less than the copyright holder’s price, etc.
- An agent refuses to pay enough to the copyright holders. Such scenarios include unauthorized agency and spreading, or concealment of all or partial sales volume by authorized agents, etc.

Infringers must somehow obtain a copy of the publication before launching any of these attacks. We have listed approaches for an infringer to obtain copies:

- **Cracking keys.** The infringer could try to steal or crack the decryption key for a copy by various means. But as cryptography improves, it is obviously costly.
- **Screen recording.** The infringer could directly obtain copies of audio, video, and images via screen recording or I/O monitoring. Defending such approaches technically seems expensive.
- **Purchasing.** The infringer could obtain a copy from the publisher by purchasing it. This approach is completely unstoppable, as the act itself is legal.

**Figure 1:**
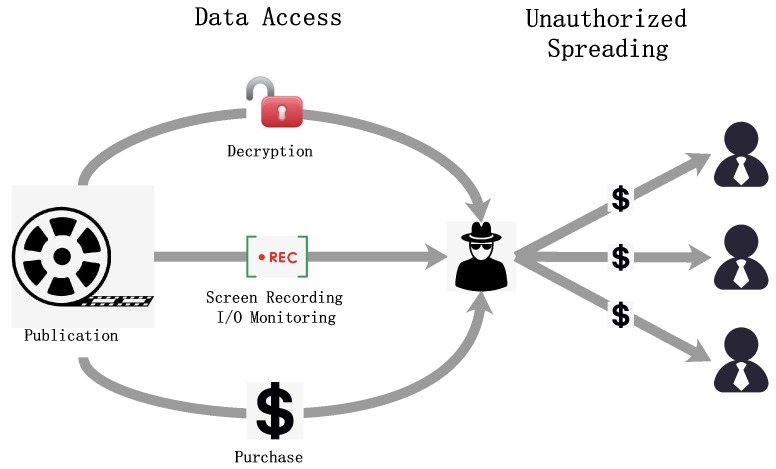
The threat model in digital rights management (DRM).

Once a copy of publication is obtained, the infringer may take profit that would otherwise belong to the copyright holder by selling the copy. The whole attack process is shown in Figure 1.

Existing mainstream or novel DRM mechanisms include but are not limited to encapsulated encryption and confusion [2,3,4,5], digital watermarks [6,7,8,9,10,11,12,13,14], blockchain [15,16], trusted computing [17,18], biological features [19], etc. After reviewing the previous schemes, we believe that existing DRM technologies still have great defects. According to the threat model and schemes mentioned above, we address some of the following challenges:

- How can we ensure that users do not actively share copies or keys? What is the methodology?
- How can we ensure that copyright holders will not bear any losses, even if users divulge copies through various means?
- How can we ensure the settlement of punishment and reward is immediate and non-repudiated?

Considering that users are selfish and greedy but rational, we try to form a competitive environment among users and amplify the cost of infringement through games. Taking advantage of the non-repudiation of smart contracts and blockchain and existing schemes related to copyright protection, we designed Y-DWMS, a YODA-based digital watermark management system.

The core idea of Y-DWMS is: under the assumption that the users are selfish and greedy but sensible, Y-DWMS infinitely amplifies the cost of infringement and rewards infringement informers in a timely manner, so as to introduce competition of interests and destroy trust among users, forcing users to opt for non-infringement and the protection of copies or keys from being cracked as a dominate decision. The design of Y-DWMS determines that users cannot bear the cost of divulging keys or copies. Technically, rewards and punishments under Y-DWMS are instantly settled and undeniable, and losses copyright holders suffer could be recovered at the moment the infringement is reported. Compared with schemes mentioned above and in Section 2, the copyright holders under Y-DWMS do not bear any losses, the informers can always get timely rewards, and the punishments of the infringers are magnified to infinity.

### 1.1. Our Contribution

In this paper, we propose Y-DWMS, a digital watermarking management system based on a public smart contract platform. We summarize the contributions of the paper as follows:

- Y-DWMS can effectively recover losses of copyright holders and track infringers, reward informers, and punish infringers at the moment of reporting, compared with [4,5,15,17,19,20].
- In the common smart contract platform, the punishment of infringers could be compensated by informers if there are malicious smart contracts signed between infringers and informers. However, Y-DWMS could resist that, compared with common smart contract platforms like [20].
- We adopt game theory to prove that Y-DWMS can increase the cost of copyright infringement to infinity and resist countermeasures by signing smart contracts. Therefore, under the deterrence of Y-DWMS, the decision of non-infringement always dominates rational users.
- Y-DWMS is independent from unique hardware, is flexible, and has weak restrictions on distribution of copies, compared with [4,5,17,19].

### 1.2. Organization of the Paper

In Section 2, we introduce representative related work and make comparisons with our scheme. In Section 3, we define the model of Y-DWMS, introduce YODA, a smart contract computing method suitable for computation-intensive contracts (CIC), and focus on the definition of system smart contracts in Y-DWMS. In Section 4, we prove that Y-DWMS could significantly amplify the cost of infringement, by adopting game analysis. In Section 5, we give the points that are still worth further discussion, and in Section 6, we give the conclusion of this paper.

## 2. Background

In this section, we organize and briefly introduce previous works on DRM and make comparisons between novel, representative schemes and Y-DWMS. The results of comparisons are in Table 1. We also propose the main technical solution of our scheme.

### 2.1. Related Work

#### 2.1.1. DRM Based on Various Encryption Techniques

The most extensively applied DRM scheme could be AACS [3], adopting many ideas like novel key encryption management, one-device-one-key, key revocation by copyright holders, and traitor tracking based on a digital watermark. Recent DRM schemes represented by Abdalla et al. [5], which are based on proxy re-encryption, address better key management in DRM schemes and can tolerate a semi-honest third party for key management. Mohamed et al. [4] improved the distribution of digital publications based on joint fingerprint decryption (JFD), and in this DRM system, each copy of the publication is encrypted differently and can only be decrypted using the corresponding purchaser key. This mechanism enhances data’s unavailability to third parties. However, even if the encryption schemes mentioned above are secure, they do not prevent users from illegally distributing copies by actively sharing their keys. Such problems are obvious in [5], and the scheme proposed in [4] fails to stop the spreading of a divulged key at the moment of its divulgence, although the scheme is claimed to be anti-collusive and traceable to traitors.

#### 2.1.2. DRM Based on Trusted Computing

In order to avoid complex key management and the unreliability of keys, some trusted computing schemes are proposed. Barbareschi et al. [17] proposed DRM system applicable to android mobile devices based on a trusted hardware core, and bind data availability to hardware; Lee et al. [19] improved DRMS based on biological features to bind data availability to a user’s fingerprint. These schemes prevented the sharing of keys, which cannot be banned by common encryption methods, and enhanced the identity-based availability of data. However, these schemes lack flexibility. Firstly, an infringer takes various approaches to illegally obtain copies, such as screen recording or I/O monitoring, or multiple people sharing a trusted device. These schemes are not defensible against such attacks. Secondly, these schemes require unique hardware, and many devices (partial PC, cable TV, etc.) currently do not support fingerprint collection or trusted platform module (TPM), which limits the generalization of the schemes above and increases costs.

#### 2.1.3. DRM Based on Blockchain and Watermarking

**Figure 2:**
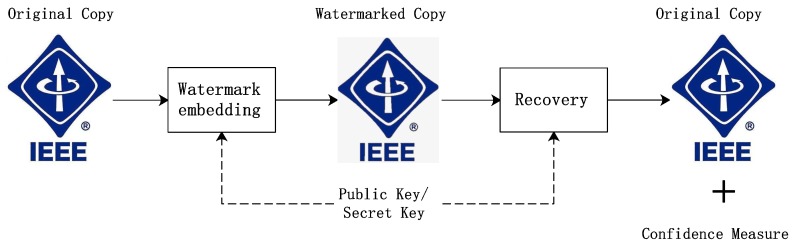
The workflow of digital watermarking.

Some people believe that protecting digital copyright by restricting the availability of copies could always be defective, and many schemes have been proposed to enhance the tracking of digital copyright infringement. Most of such schemes adopt the digital watermark as a basic technique to embed digital signatures in publications. Figure 2 shows the workflow of digital watermarking. The DRM system represented by the scheme proposed by Ma et al. [15] embeds information, including the identities of purchasers, into digital copies and manages it with blockchain, so as to solve the problem of screen recording. The scheme proposed by Hasan et al. [20] adopts smart contracts to ensure the process of trade between untrusted users and novel techniques like IPFS [21] to ensure the integrity of digital assets. However, the limitations of such schemes still remain. Firstly, the drawback of not being able to prevent the offline spread of the divulged copies still exist. Secondly, independently collecting evidence by platform could be difficult, but the users’ motivation for reporting is not well enhanced as such schemes lack an effective punishment and reward mechanism. As a result, the scheme mentioned above is powerless to track the traitor in a timely manner. In addition, the scheme proposed in [20] cannot resist the countermeasure of signing a smart contract between the infringer and informer, which eliminates the punishment the infringer deserves.

### 2.2. Our Solutions

After reviewing previous schemes, we believe that existing DRM technologies still have great defects. Considering that users are selfish and greedy, but rational, we try to form a fierce competitive environment among users and amplify the cost of infringement through games. Taking advantage of the non-repudiation of smart contracts, non-tampering of blockchain, and existing schemes related to copyright protection, we designed Y-DWMS according to following ideas.

- In order to prevent users from sharing encryption keys or accounts, we use a public wallet key for users to control the copy encryption and account login, trying to bind key and account safety to the property safety of users, which amplifies the cost infinitely.
- In order to secure digital rights under the worst condition where an infringer has divulged the copy, a punishment and reward mechanism based on smart contracts is designed to verify watermarks in the divulged copy, authenticate the informer’s report, trace the infringer, perform punishment, reward informers, and recover losses the copyright holders suffered.

## 3. Our Scheme

In this section, we propose a model of Y-DWMS based on the threat model above and our strategy, attempting to achieve an effective game-theoretical copyright management system. Before elaborating on Y-DWMS, we give some definitions and symbols that will be used in future designs.

### 3.1. Definitions

#### 3.1.1. Symbols

As shown in Table 2, we give symbols and their meanings. These symbols will appear in following descriptions.

#### 3.1.2. YODA: Enabling Computationally Intensive Contracts

The execution of some computationally intensive contracts (CICs) is extremely expensive, which prevents us from performing digital watermarks and complex encryption algorithms at low gas costs on smart contract platforms like Ethereum. Selecting a small set of miners performing the calculation of contracts could reduce costs, but it does not guarantee credibility of the execution. Sourav Das et al. proposed YODA [22], a method for deploying CICs on Ethereum. YODA proposed a novel multi-round adaptive consensus using likelihood estimation (MIRACLE) algorithm based on a sequence hypothesis test to execute the smart contract: a quite small number of miners are pseudo-randomly selected to form an execution set ES, in which each miner executes the contract separately to perform a single round calculation. MIRACLE executes CICs through multiple round iterations in different ES each round and decides the result through likelihood estimation. This paper also proposed a novel **randomly inserted contract execution** (RICE) algorithm to force miners to perform CICs correctly. RICE’s logic is as follows:

**Initialization.**Ψ is a smart contract operating on a state denoted as σ, taking *x* as input. Ψ(σ, *x*) outputs a new state denoted as $\sigma^{*}$. Denote root(σ) as a hash digest of σ and *j* as a round number MIRACLE has executed. In order to ensure that the pseudo-random abstracts are generated in each round without breaking the mapping relationship between them, RICE generates a digest denoted as (seed(j,.),root), in which seed(j,.) is generated like:

$${\mathrm{seed}}^{\left(j,0\right)}\leftarrow \left\{\begin{array}{cc} \;\mathrm{RandomGen}\;() & \;\mathrm{if}\;j=1,\\ \;\mathrm{hash}\;(\mathrm{seed}{\;}^{\left(j-1,0\right)}) & \;\mathrm{otherwise}.\; \end{array}\right.$$

**CIC Execution.** Considering the machine instruction sequence of a smart contract as a vector, RICE updates seed at a pseudo-random position of this vector. Denote the state when updating seed as $\sigma^{\prime }$; RICE updates seed like:

$${\mathrm{seed}}^{\left(j,l+1\right)}\leftarrow \mathrm{hash}\left({\mathrm{seed}}^{\left(j,l\right)}∥\mathrm{root}\left(\sigma^{\prime }\right)\right)$$

This ensures that each round of CIC execution generates a different digest.

**Result Submission.** After execution, miners in ES submit (seed(j,Φ), root($\sigma^{*}$)) via a consensus protocol, where ϕ is the number of seed updates. Seed generated by honest miners should be the same in a round but different between rounds. Dishonest execution leads to mismatching between root and digest.

Although YODA is not directly reflected in the contract logic of Y-DWMS, as scheme for Y-DWMS to execute smart contracts, YODA not only enables smart contracts to perform computationally intensive calculations such as digital watermarks at low cost, but also ensures that it is difficult for adversaries to collude with miners to carry out wrong results. We could isolate, trace, and punish the malfunctioning miners in time. Chainspace [23], proposed by Al-Bassam et al. is a smart contract infrastructure that can implement YODA well. The mapping requests to ES and the isolation of malfunctioning miners are implemented by the system smart contract.

Here, we formalize the interaction process of YODA:

**Propose.** User generates request package R, and submits it to AN. AN broadcasts R to the whole network.

**Generate ES.** Pseudo-randomly chosen miner N${}_{i}$ joins ES, which makes ES={N${}_{i}$∣ i = SysContract.RandomSelection(R)}.

**Execution.** For each Ni∈ES, independently execute C${}_{R}$, generating (R, C${}_{R}$, Sig${}_{N_{i}}$, S&R${}_{i}$).

**Consensus In ES.** For each N${}_{i}$∈ ES, broadcast T${}_{N_{i}}$ = (R, C${}_{R}$, Sig${}_{N_{i}}$, S&R${}_{N_{i}}$) to other miners in ES. ES reaches T${}_{ES}$ through a consensus protocol like PBFT. Broadcast T${}_{ES}$ to ¬ES. Generate new ES’ and re-execute C${}_{R}$.

**Verification & Serialization.** ¬ES maintains O = {T${}_{1}$, T${}_{2}$, …} and determines the final result from O using likelihood estimation. ¬ES serializes the result and sends feedback to ES to terminate the calculation.

#### 3.1.3. Pivotal Contracts

Specifically, the basic function of Y-DWMS is implemented through pivotal smart contracts. After receiving requests, a node’s core module processes the request and executes one of the following contracts. In Y-DWMS, there are two pivotal smart contracts:

**Publish.** This contract is released when copyright holders decide to publish a publication and is invoked when a user applies to purchase a copy. A user transfers a specific amount of deposit to this contract’s account, submitting a request meanwhile. After verification of the signature, a user’s account will be added to a list, the users in which have already paid for the copy. Copyright holders can inspect the current status of the list, embed their signatures and the user’s signature to each copy, and distribute it to the related user. If the copyright holders decide to destroy the contract, the deposit stored in this contract will be settled to the copyright holders, and the process of publishing will be terminated. This contract’s logic is as Algorithm 1.  

**Algorithm 1** Contract_Publish
1:**Var**:2:{account, cp_holder, customer:*Address*; paid_list:*Address* list;}3: 4:**Func_Purchase()**:5:**if** exists transaction{customer->account, amount=deposit} **then**6: paid_list.add(customer)7:
**else**
8: reject and fallback9:
**end if**
10: 11:**Func_Distribute(publication)**:12:publication<-Watermark(publication, customer.SK, cp_holders.SK)13:publication<-Encrypt(publication, customer.PK)14:paid_list.remove(customer)15:**return** publication16: 17:**Func^~^**:18:**if** paid_list **is** NULL **then**19: account.transfer(cp_holder, amount)20:
** destroy**
21:
**else**
22: reject and fallback23:
**end if**



**Report**. This contract is released when copyright holders decide to publish a publication and is invoked when a user reports an infringement. After a user transfers a deposit to Contract_Publish, and before further processes, the copyright holder should ask the user to transfer a specific amount of deposit to this contract. An informer submits a copy, which is purchased and divulged by an infringer, and a parameter *X* to this contract, and if the infringer’s watermark is detected, this contract will transfer a deposit of *X* to the informer and deposit copyright holders deserve to copyright holders. The logic of this contract is as Algorithm 2.    

**Algorithm 2** Contract_Report

1:**Var**:2:{account, cp_holder, customer, informer:*Address*; X, price:*Number*; pub:*Binary*;}3: 4:**Func_Report()**:5:confidence<-WatermarkVerify(customer.PK, cp_holder.PK, pub)6:**if** confidence **is** True **then**7: **if** X+price<=account.deposit **then**8:  account.transfer(informer, X)9:  account.transfer(cp_holder, price)10:  X<-011: **else**12:  X<-X-account.deposit13:  account.transfer(cp_holder, price)14:  account.transfer(informer, account.deposit)15: **end if**16:
**else**
17: reject and fallback18:
**end if**
19: 20:**Func^~^**:21:**if** X **is** 0 **then**22: account.transfer(customer, account.deposit)23: **destroy**24:
**else**
25: reject and fallback26:
**end if**




### 3.2. System Modeling

**Figure 3:**
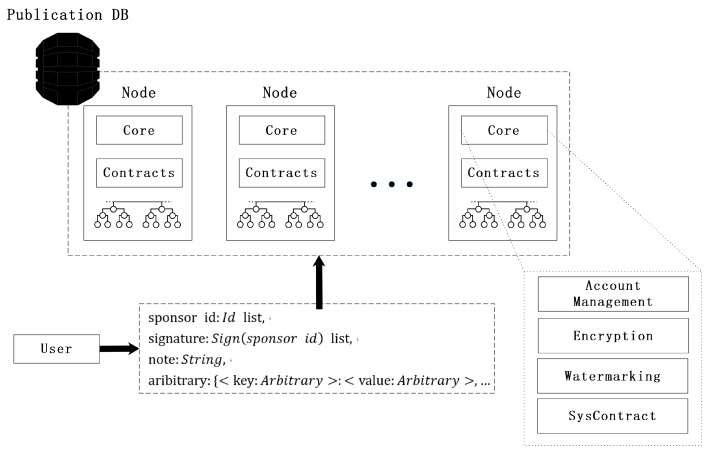
System architecture of Y-DWMS.

Y-DWMS takes a distributed network as its infrastructure, and each node runs three modules: a **ledger module**, a **core module**, and a **contract module**. In the case that the number of adversarial nodes meets the worst case, 3f+1<n, an appropriate consensus protocol guarantees the final consistency of each node state. The system overview is shown in Figure 3.

The **ledger module** is a distributed blockchain ledger that stores a history of operations, records of purchasing, using, and downloading, as well as payment and contract records between users. As the attribute of the blockchain, transactions cannot be modified once they are serialized on the blockchain.

The **core module** is the module responsible for sharing and contract execution. In this module, we deploy the entire YODA algorithm to manage the status of contracts. Nodes are required to have high integrity. Every node newly added to the network must accept the integrity inspection from other nodes through the consensus protocol, and other nodes decide admission independently according to the integrity of new nodes.

The **contract module** manages all smart contracts and invokes them according to a user’s request, controlled by the core module. The smart contract takes requests as input, operates on a contract’s status, and provides feedback to the corresponding user. The smart contract implements business logic, and its ideal scripting language is with Turing completeness. The execution of a smart contract is atomic.

**Requests** are generated by users to interact with the platform. Users define various key–value pairs in a set of requests to express business logic to the platform. The platform classifies the requests, calls the corresponding smart contracts to process user requests, and serializes transactions on the ledger. The data structure of a request is as follows:

$$\begin{array}{c} \;\mathrm{typedefine}\;<\mathrm{type}>:\;\mathrm{Request}\{\\ \;\;\mathrm{sponsor}\;\mathrm{id}:\;\mathrm{Id}\;\mathrm{list},\;\\ \;\mathrm{signature}:\;\mathrm{Sign}(\mathrm{sponsor}\;\mathrm{id})\;\mathrm{list},\;\\ \;\mathrm{timestamp}:\;\mathrm{Timestamp},\;\\ \;\;\mathrm{note}:\;\mathrm{String},\;\\ \;\mathrm{aribitrary}:\;\{<\mathrm{key}:\;\mathrm{Arbitrary}>:<\mathrm{value}:\;\mathrm{Arbitrary}>\}\\ \;\} \end{array}$$

#### Interaction

**Figure 4:**
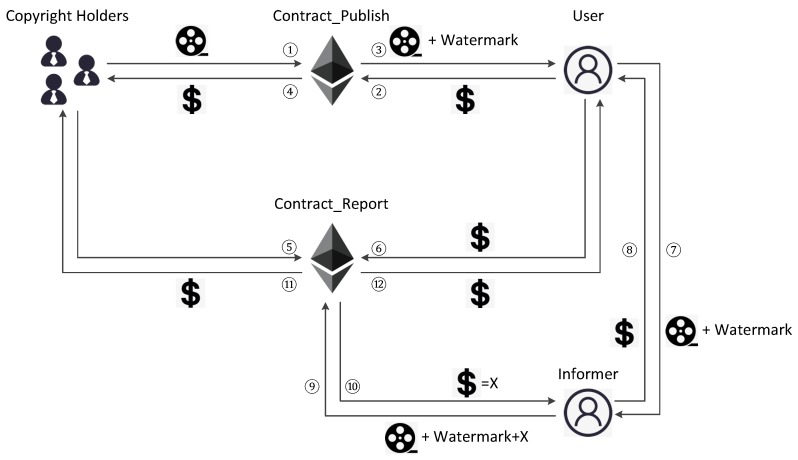
Process of interaction of Y-DWMS.

We will present the full workflow and process of interaction step by step. The interaction of Y-DWMS is as simple as the common smart contract platforms like Ethereum: copyright holders release smart contracts to gather and manage deposits from users, and deposits will be transferred to specific accounts if specific transactions occur. The process of interaction is presented in Figure 4.

- Step ➀: The copyright holders release Contract_Publish and store publication to this contract.
- Step ➁: A user who decides to purchase stores deposits to this contract.
- Step ➂: Contract_Publish returns a copy of the publication embedded with copyright holder and user watermarks.
- Step ➃: When Contract_Publish is destroyed, copyright holders receive a deposit.
- Step ➄: Meanwhile, copyright holders release Contract_Report, which is aimed to the user, and ask the user to store a deposit to this contract.
- Step ➅: The user stores a deposit to this contract.
- Step ➆: The user divulges the copy by selling it to an informer.
- Step ➇: The informer pays for the divulged copy.
- Step ➈: The informer reports the infringement by submitting the copy and parameter *X* to Contract_Report.
- Step ➉: If a watermark is detected in the copy and verified by Contract_Report, the informer will get a deposit, the amount of which is *X*.
- Step ⑪: Meanwhile, copyright holders will get the deposit they deserve from publishing the publication.
- Step ⑫: If there is no infringement during a period that copyright holders consider appropriate, the deposit will be returned to the user.

## 4. Analysis Combining Game Theory

Game theory is an important theoretical tool, supporting many solutions like those in [24]. In this section, we sort out all possible means of infringement and prove the incredible cost that the infringer needs to bear through game analysis. We find that in a full game, no infringement always dominates a user’s decision.

### 4.1. Copyright Offense by Colluding with Miners: Game Theoretical Dangerous

According to YODA’s design, an adversary needs to calculate the ES of each round of execution before submitting an illegal request and force a significant number of miners in each ES to perform the wrong calculation. If this fails, the request is rejected, and the miner will be isolated at a cost considered to be −∞. The MIRACLE and RICE algorithms ensure that the processing of a request is repeated for multiple rounds by multiple random ES, and the calculation process cannot be forged. The feasibility and security of YODA were respectively proved [22], so we can assume that the probability of attack failure β is close to 1. The value of the publication is denoted as *v*, the number of miners in ES is denoted as *n*, and the cost of controlling a miner is expressed as *c*. Then the profit expectation of this process is

$$Ex=\lim_{\beta \to 1}v-\beta \infty -\left(1-\beta \right)nc=-\infty .$$

If copyright payment is regarded as value exchange, we can assume that the user’s profit in this process is 0. The user’s profit matrix can be obtained:

Non-infringementInfringement0
$\lim_{\beta \to 1}v-\beta \infty -\left(1-\beta \right)nc$


Obviously, in this single-player static game, non-infringement decision forms dominance. Therefore, performing copyright infringement via collusion with miners is almost impossible, such an attempt is also extremely costly.

### 4.2. Copyright Offense via Sharing Publication Copy: Game Theoretical Dangerous

One common way to circumvent Y-DWMS and other DRM schemes is to share copies outside the jurisdiction of the platform. Assuming that the infringer has already obtained and divulged copies through screen recording or I/O listening, the following subgame is introduced.

**Figure 5:**
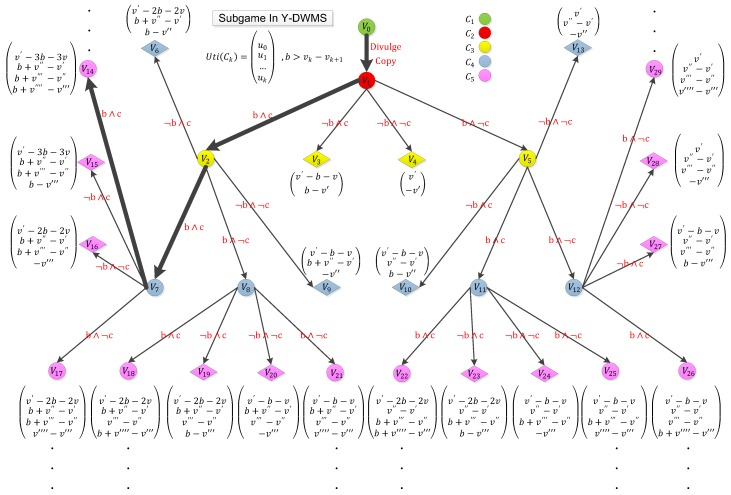
The subgame will be triggered if a digital copy is divulged.

In the game presented in Figure 5, the player set contains all users and the copyright holder, i.e., N = {holder, C${}_{1}$, C${}_{2}$, …, C${}_{k}$}. For each user C${}_{k}$, the action set is A = s ∧ r, ¬ s ∧ r, s ∧¬ r, ¬ s ∧¬ r, in which selling copies is denoted as *s*, and reporting is denoted as *r*. To simplify the analysis, we firstly assume that each user is allowed to perform selling copies only once, and the copyright holder’s profit will not be discussed in figures (we will discuss these respectively). We denote the profit function of C${}_{k}$ as u${}_{k}$.

Next, we perform the analysis of this game. According to the contract design of Y-DWMS, this game adopts the following rules:

- If C${}_{k}$ launches s, then the profit of C${}_{k}$ increases by v${}_{k}$, and the profit of C${}_{k+1}$ increases by –v${}^{k}$. v${}^{k}$ is the price for selling divulged copies.
- If C${}_{k}$ launches r, then the profit of C${}_{k}$ increases by b, the profit of C${}_{1}$ increases by –b–v, and the profit of the copyright holder increases by v. b is the reward for C${}_{k}$, and v is original price of the publication.

If *N* is an infinite set, i.e., the number of users is large enough, the deposit the infringer transferred to the contract is sufficient, and b > v${}_{k}$−v${}_{k+1}$, then the whole game will form a unique sequential dominance. For C${}_{2}$, C${}_{3}$, …, C${}_{k}$, the choice of s∧r will always bring the highest profit, and the dominance sequence will always be the leftmost route of the game tree. This game ideally will not terminate since N is an infinite set.

Formally, we have the followings:

If N is an infinite set, the balance of the user’s account is sufficient and b > v${}_{k}$−v${}_{k+1}$, then the subgame in the figure will perform a unique sequential equilibrium

$$Ex=\lim_{k\to +\infty }\left(s_{1},s_{2},...,s_{k}\right),\left(b_{1},b_{2},...,b_{k}\right),$$

where

$$\left\{\begin{array}{c} s_{1}=\left(\left[1\left(Divulge\;Copy\right)\right]\right)\\ s_{2}=\left(\left[1\left(s\wedge r\right),0\left(\neg s\wedge r\right),0\left(s\wedge \neg r\right),0\left(\neg s\wedge \neg r\right)\right]\right)\\ s_{3}=\left(\left[1\left(s\wedge r\right),0\left(\neg s\wedge r\right),0\left(s\wedge \neg r\right),0\left(\neg s\wedge \neg r\right)\right]\right)\\ \ldots \\ s_{k}=\left(\left[1\left(s\wedge r\right),0\left(\neg s\wedge r\right),0\left(s\wedge \neg r\right),0\left(\neg s\wedge \neg r\right)\right]\right)\\ b_{1}=\left(\left[1\left(v_{0}\right)\right]\right)\\ b_{2}=\left(\left[1\left(v_{1}\right)\right]\right)\\ b_{3}=\left(\left[1\left(v_{2}\right),0\left(v_{3}\right),0\left(v_{4}\right),0\left(v_{5}\right)\right]\right)\\ b_{4}=\left(\left[0\left(v_{6}\right),1\left(v_{7}\right),0\left(v_{8}\right),0\left(v_{9}\right)\right]\right)\\ b_{5}=\left(\left[1\left(v_{14}\right),0\left(v_{15}\right),0\left(v_{16}\right),0\left(v_{17}\right)\right]\right)\\ \ldots \\ b_{k}=\left(\left[1\left(v_{l2}\right),0\left(v_{l1}\right),0\left(v_{r1}\right),0\left(v_{r2}\right)\right]\right). \end{array}\right.$$

Under this equilibrium, the profit of C1 is

$$Uti\left(C_{1}\right)=\lim_{k\to \infty }v^{\prime }-k\left(b+v\right)=-\infty$$

when the profit of copyright holder is

$$Uti\left(Holder\right)=\lim_{k\to \infty }kv.$$

Obviously, the cost of C${}_{1}$ is incredible, while the copyright holder suffered no loss.

**Figure 6:**
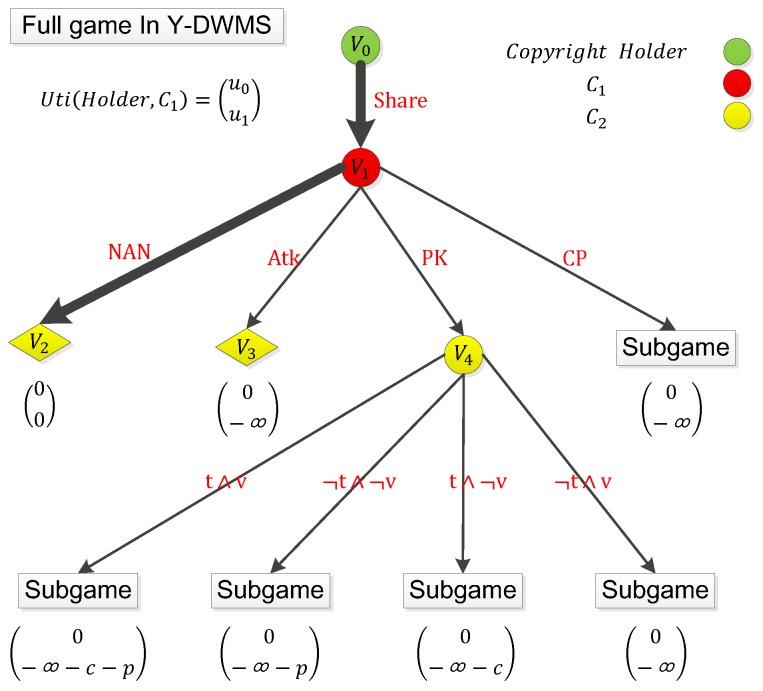
The full game in Y-DWMS. Sharing copies will always trigger the subgame.

Based on this subgame, the benefit of the infringer divulging the key is easy to calculate: instead of generating additional positive profit for C${}_{1}$, it adds negative revenue –c–p, where c is the property loss C${}_{1}$ needs to suffer, and p is the losses generated by other users stealing C${}_{1}$’s identity. Based on the previous profit analysis of various attack means, we could perform the full game in Figure 6:

In the full game, the player set contains all users and the copyright holder, i.e., N = {holder, C${}_{1}$, C${}_{2}$, …, C${}_{k}$}. The action set of C${}_{1}$ is A = {Atk, CP, PK, NAN}, where Atk is a direct attack on Y-DWMS, CP is the divulged copy, PK is divulged public key of C${}_{1}$, NAN is nothing to do. The action set of C${}_{2}$ is A’ = {t ∧ v, ¬ t ∧ v, t ∧¬ v, ¬ t ∧¬ v}, where t is stealing C${}_{1}$’s balance, and v is stealing C${}_{1}$’s identity. To simplify the analysis, we only discuss the profit of the copyright holder and C${}_{1}$, and the matching profit function is u${}_{0}$, u${}_{1}$. According to the design of contracts, the full game adopts new rules on the basis of a subgame:

- If C${}_{2}$ launches t, then the profit of C${}_{1}$ increases by –c, profit of C${}_{2}$ increases by c.
- If C${}_{2}$ launches v, then profit of C${}_{1}$ increases by –p.

According to our game analysis of the infringer launching an attack on Y-DWMS, the profit of C${}_{1}$ launching Atk is −∞. No matter whether C${}_{1}$ launches CP or PK, the subgame will be triggered. More importantly, launching PK generates additional negative profit. It is obvious that we have the following:

If the full game satisfies the conditions satisfied by the subgame, then a unique sequential equilibrium will form, where

$$\left\{\begin{array}{c} s_{1}=\left(\left[1\left(Share\right)\right]\right)\\ s_{2}=\left(\left[0\left(Atk\right),0\left(CP\right),0\left(PK\right),1\left(NAN\right)\right]\right)\\ b_{1}=\left(\left[1\left(v_{0}\right)\right]\right)\\ b_{2}=\left(\left[1\left(v_{1}\right)\right]\right). \end{array}\right.$$

Under this equilibrium, the profit of C${}_{1}$ is 0 when the profit of the copyright holder is still $\lim_{k\to \infty }kv$.

### 4.3. Countering Punishment via Smart Contract: Impossible

There is a fatal weakness in traditional smart contract schemes that an infringer could adopt to eliminate punishment, by forcing users to sign a smart contract before purchasing copies from the infringer. We denote this as **Contract_Countermeasure**. If the copies are submitted to the platform and the infringer’s watermark is detected, this contract is triggered to transfer a huge amount of compensation to the infringer, which is denoted as cmp. Thus, if the infringement is reported, the informer will suffer heavy losses. The contract’s logic is simply presented as Algorithm 3.

**Algorithm 3** Contract_Contermeasure

1:**Var**:2:{account, infringer, informer:*Address*;}3: 4:**Func_Contermeasure()**:5:**if** exists transaction{Contract_Report->informer, amount=deposit} **then**6: account.transfer(infringer, deposit)7:
**else**
8: reject and fallback9:
**end if**
10: 11:**Func^~^**:12:
**destroy**




**Figure 7:**
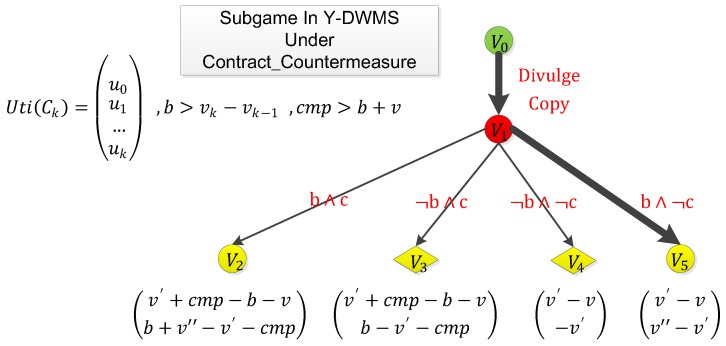
The subgame mentioned above will be changed like this under **Contract_Countermeasure**.

Under a contract like this, the subgame mentioned above will be changed as in Figure 7.

However, in Y-DWMS, informers can counter it simply. As an informer reward request is a secret parameter that the infringer will never correctly predict, this contract cannot know the exact loss the infringer will suffer. The compensation to the infringer must be settled when **Contract_Countermeasure** is signed, so the informer could simply increase the reward request, making sure that $b>v^{\prime }+cmp$. As a result, the domain decision will switch to b∧c, maintaining the original subgame and full game.

### 4.4. Summary

Analysis above proves that in Y-DWMS, losses infringer suffers through common methods of copyright infringement could always be unbearable, and the copyright holder does not bear any loss. Under the control of smart contracts, the settlement of profit is instantaneous and undeniable. This minimizes the influence of psychological factors on user’s decision. Under the condition of complete information, rational and self-interested users will make efforts to protect the copies they occupied from being stolen, and steal keys and copies of others. Therefore, a fierce competition is introduced among users. We also introduced a counter contract which is effective in common smart contract platform, and proved that such countermeasure is ineffective in Y-DWMS.

## 5. Discussions

Y-DWMS integrates the advantages of various current anti-infringement schemes, with a simple structure and implementation. However, as Y-DWMS is still in an early stage of development, by combining smart contracts with digital watermarks, there are some security issues that need to be addressed and optimized [25]. Before Y-DWMS becomes an effective system, the following security issues need to be discussed in depth:

**Account Security.** Admittedly, the credible and efficient game environment provided by Y-DWMS can effectively restrain infringement in advance in a flexible way, but this environment is built on the basis of secured accounts. If malicious users crack other users’ keys or copies through technical means, the stolen users will bear the risk of gaming, and the proof and traceability of such an infringement is extremely difficult. Therefore, the effective premise of Y-DWMS is that the user’s account security is fully guaranteed.

**Loopholes in Ethereum.** The design of Y-DWMS is succinct and clear, so it will not bring about fatal system loopholes to the scheme. However, practically, there are still security issues that need to be considered.

Security issues are obvious on common smart contract platforms, Ethereum being a representation. Ethereum is facing security threats including but not limited to loopholes caused by less rigorously programmed smart contracts, DAO loopholes. The model of Y-DWMS relies on an ideal smart contract platform, and without fixing the loopholes mentioned above, the effectiveness of Y-DWMS will decay. However, a methodology of securing network infrastructure and the smart contract platform could be a tough task.

**Security Issues with Digital Watermarks.** Y-DWMS adopts ideal digital watermark algorithms, which is robust: even if watermarks are damaged or compressed to an extent, the algorithms could still detect and verify watermarks. This puts forward higher requirements for digital watermark algorithms. Many digital watermark algorithms are claimed to be perfect in their performance, but the robustness, efficiency, and implementation difficulty of such algorithms have yet to be proved.

**Privacy.** Most smart contract applications, including Y-DWMS, face privacy issues, as there are conflicts between privacy demand and the transparency of blockchain and smart contracts. In Ethereum, anyone could inspect the current status of smart contracts, which also contains information about personal consumption. As for Y-DWMS, the informer could inspect the contract status to access history of consumption, which should have been private. An effective access control system on smart contracts plays a significant role in solving the conflicts mentioned above. Deploying Y-DWMS on a smart contract platform like Hyperledger, which has a better access control system, should be considered.

## 6. Conclusions

In order to address the divulgence of digital publication copies and immediate compensation of losses copyright holders suffer, in this paper, we proposed Y-DWMS, a YODA-based digital watermark management system, to counter the mainstream illegal distribution of publications. The main conclusions are as follows:

- Adopting smart contracts and digital watermarks could form a game between infringers and informers, as divulged copies are embedded with the signatures of infringers, and informers could get profit from infringers by submitting signed divulged copies to smart contracts. Losses copyright holders suffer could also be recovered by smart contract.
- Game analysis has proved that the game mentioned above could theoretically amplify the cost of infringement to infinity, under the condition that the deposit of the infringer is sufficient, as the distribution of the divulged copy is unstoppable among an infinite set of users under such a game environment.
- Game analysis has also proved that the punishment infringers deserve could not be recovered by signing smart contracts, as the logic of Contract_Report makes infringers never know how much deposit will finally lose.

Y-DWMS theoretically prevents digital rights infringement fundamentally, with high flexibility, efficiency, and credibility. We need to consider implementing a secure smart contract platform as an important future work, as Y-DWMS takes such a platform as the basis of its effectiveness. We believe that Y-DWMS provides new thinking for further research on solving digital copyright issues.

## Figures and Tables

**Table 1:** Comparison of different schemes and our YODA-based digital watermark management system (Y-DWMS).

	Y-DWMS	Abdalla et al. [5]	Mohamed et al. [4]	Barbareschi et al. [17]	Lee et al. [19]	Ma et al. [15]	Hasan et al. [20]
Heavy cost of infringe-ment	*√*	×	×	×	×	×	×
Recovery of copyright losses	*√*	×	×	×	×	×	×
Undeniable reward and punishment	*√*	×	×	×	×	×	×
Tracking traitors	*√*	×	*√*	*√*	*√*	*√*	*√*
Screen record prevention	*√*	×	×	×	×	*√*	×
Independent from uni-que hardware	*√*	*√*	*√*	×	×	*√*	*√*

**Table 2:** Symbols and meanings in the protocol.

Symbols and Their Meanings	
R	Request package generated by user
C${}_{R}$	Smart contract corresponds with R.
ES = {N${}_{1}$, N${}_{2}$, N${}_{3}$…}	A small pseudo-random set of executions where miners execute C${}_{R}$ independently, ES is redefined after each round of MIRACLE.
O = {T${}_{1}$, T${}_{2}$, T${}_{3}$…}	Result set, saving results of each round of MIRACLE execution. The final result of C${}_{R}$ is selected from O.
AN	Anchor node for the communication between ES and ¬ES
N	Miners
T	Tetrad of result
Sig${}_{N}$	Signature of miner N
S&R	$(seed^{\left(j,\Phi \right)}$, root($\sigma^{*})$), representing result of RICE.
